# Alternative endpoints to mortality in cancer screening trials

**DOI:** 10.1002/1878-0261.13697

**Published:** 2024-07-06

**Authors:** Sir Harpal Kumar

**Affiliations:** ^1^ BioPharma Business & Europe GRAIL Bio UK Ltd. London UK

**Keywords:** alternative endpoints, cancer screening, early detection of cancer, liquid biopsy

## Abstract

The reliance on mortality endpoints in cancer screening trials is not always compatible with the need to accelerate progress in outcomes for patient and public benefit. Evaluation of novel cancer screening technologies, such as multi‐cancer early detection (MCED) tests, could be expedited by using alternative metrics that are measurable earlier than mortality. These include endpoints based on cancer stage at diagnosis, such as reduction in late‐stage cancer incidence, and endpoints following cancer diagnosis, such as eligibility for curative therapy. Innovative trial designs with earlier measures that complement cancer mortality are needed to realise the potential benefits of novel screening technologies such as MCEDs more rapidly.

## Graphical abstract

The reliance on mortality endpoints in cancer screening trials is not always compatible with the need to accelerate progress in outcomes for patient and public benefit. Innovative trial designs with earlier measures that complement cancer mortality are needed to realise the potential benefits of novel screening technologies such as multi‐cancer early detection (MCED) tests more rapidly.

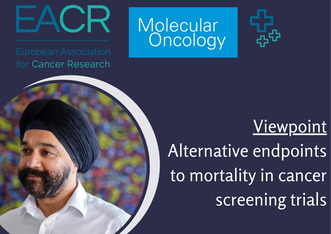

**Abbreviations**
HICs: high‐income countries
MCED: multi‐cancer early detection
NSC: UK National Screening Committee
RCT: randomised controlled trial
USPSTF: US Preventive Services Task Force

## 1 Introduction

Cancer is the leading cause of death in high‐income countries (HICs) [1]. The World Health Organization recently predicted that nearly 11 million new cancer cases will occur in HICs in 2050, a 37% increase from the 8 million estimated in 2022 [2]. The vast majority of cancer deaths are associated with late stage at diagnosis, usually after patients present with symptoms. Detecting cancer at an early stage, and ideally prior to symptomatic presentation, when there are more treatment options and greater potential for cure, can help reduce this unacceptably high cancer burden. It is therefore imperative that patients benefit from new, safe, and effective technologies for earlier asymptomatic cancer detection.

Historically, the sequence of evidence generation for cancer screening tests has taken decades, from development and validation of a new technology, through clinical trials assessing utility and publication of their effectiveness, to implementation at population scale. One reason for this prolonged time frame is the requirement for evidence of a reduction in cancer mortality, which necessitates observation of very large study populations and up to 20 years of follow‐up. Cancer's rising human and economic toll demands a sense of urgency as we consider novel approaches to screening.

The advent of multi‐cancer early detection (MCED) brings unique challenges and opportunities for evaluating the benefits and harms of screening. Given the rapid pace of technological development in blood‐based assays, sequencing, and artificial intelligence, a new and rigorous approach applicable to the MCED paradigm is required to efficiently assess its effectiveness [3]. The use of outcomes that are measurable earlier than mortality, such as reduction in late‐stage cancer incidence [4], could meaningfully expedite the read‐out of trials and thus progress in cancer screening.

## 2 The expectation, the reality, and the alternative

In response to the surge in biomarker research in the late 1990s, a blueprint was established to standardise the phases of screening test development, from discovery through to randomised controlled trial (RCT) [5]. This blueprint specified a statistically significant disease‐specific mortality benefit as the primary endpoint and indicator of screening effectiveness in the RCT phase. Mortality captures the collective contributions of screening, diagnosis and treatment, but it does not address the additional benefits of early cancer detection relevant to treatment eligibility, functional status, cost of care or patient reported outcomes. It is also unclear whether any cancer screening biomarker discovered since publication of the blueprint has been through all phases of the prescribed development process.

The ultimate goal of cancer screening is to reduce the morbidity, mortality and cost to individuals and to society of late‐stage cancer. Decision‐making bodies such as the UK National Screening Committee (NSC) and the US Preventive Services Task Force (USPSTF) make recommendations for a screening modality based on the balance of its benefits and harms. All screening tests have the potential to do harm as well as good, not necessarily due to the tests themselves, but often due to the downstream consequences. Rigorous evidence should always be a prerequisite for screening implementation, especially for population‐based programmes in predominantly healthy people. However, a reliance on mortality endpoints in screening RCTs is not compatible with the need to expedite progress for patient and public benefit. Indeed, screening for breast, cervical and colon cancer was implemented in some countries before studies with observed mortality outcomes were completed. It is therefore worth asking the question why a substantially higher evidence standard should be applied to newer screening tests. The opportunity cost of delaying implementation should be weighed carefully against the evidence available at a given time.

In an average‐risk population, mortality related to a single cancer type is low. Demonstrating a mortality benefit therefore requires large and lengthy screening RCTs. For uncommon cancer types, this delay and uncertainty is compounded. It is highly unlikely that a single cancer screening test for an uncommon cancer type could ever meet the traditional evidence bar for recommendation, due to low mortality, consequent lack of statistical power in large trials, and lack of cost‐effectiveness. Together, however, these cancers comprise a large proportion of the total cancer burden—roughly a quarter of cancer cases in the UK and Europe each year, and an even larger proportion of deaths [6]. The simultaneous detection of uncommon cancer types with no established screening modality is a major strength of MCED tests. However, they require trial endpoints to be assessed at the aggregate, not single cancer, level. This creates further challenges for a mortality endpoint given the variable mortality across cancer types, most likely necessitating even larger and longer trials than any single cancer screening test to have sufficient power.

Over the 10–20 years it takes to assess mortality outcomes in a conventional RCT, advances in the genomics‐based technologies and bioinformatics used in an MCED test are likely to be more rapid than those in the imaging or endoscopic technologies used in most single cancer screening programmes. Such advances may outpace the screening technology under study, potentially rendering the test itself obsolete. Furthermore, continued improvements in cancer diagnosis, treatment, and survival in this time frame may also make it difficult to disaggregate the specific contribution of the screening intervention on cancer mortality from these other, potentially pivotal, contributions, and/or reduce the relevance of the screening intervention to contemporary practice. Indeed, much of the benefit seen in breast cancer mortality since the advent of breast screening has been in the innovation and development of new breast cancer treatment paradigms for early‐stage disease, which were made possible by the fact of being able to detect these cancers at earlier stages. Even trends in cancer incidence and stage at diagnosis can change significantly over long timescales, impacting trial design parameters such as statistical power and effect size.

For all these reasons, alternative trial endpoints are needed to expedite the evaluation of novel MCED screening tests. Endpoints based on reductions in late‐stage cancer incidence have been proposed as early indicators of screening effectiveness [7]. Models are commonly used to project the mortality benefit of early detection tests [8, 9], and a strong association between reduction in late‐stage cancer incidence and reduction in long‐term cancer‐specific mortality has been shown across a number of large cancer screening RCTs for lung (*R* ^2^ = 0.79 based on three recent large trials and 0.996 based on 12 trials), breast (*R* ^2^ = 0.94) and prostate cancers (*R* ^2^ = 0.98), and colorectal cancer based on metastatic disease (*R* ^2^ = 0.93) [10]. Weaker associations have been shown between reduction in late‐stage cancer incidence and short‐term mortality [11].

Cancer outcomes are determined by a variety of factors including diagnostic and treatment interventions, which vary by cancer type. Thus, the achievable mortality reduction from a given reduction in late‐stage cancer is also expected to vary by cancer type. However, the strong concordance between late‐stage cancer incidence and subsequent mortality for lung, breast, prostate, and colorectal cancers suggests that late‐stage cancer incidence could be a reliable early indicator of cancer‐specific mortality across the many cancer types detected by MCED screening [10]. Notably, almost all observed reductions in cancer mortality as a result of screening have been preceded by a reduction in late‐stage diagnosis of that cancer type. By corollary, if a reduction in late‐stage diagnosis is not observed in an RCT, this may indicate a low likelihood that the screening test will ultimately yield a mortality benefit. Standard examples of ‘failed screening’, such as for thyroid cancer and neuroblastoma, would not have met the reduced late‐stage cancer incidence criterion in an RCT.

Computational disease modelling plays a key role in extrapolating learnings from the available evidence. These models can be powerful tools for predicting more distant endpoints from related proximate ones, such as expected mortality reduction from a given reduction in late‐stage cancer, and may even have advantages over conventional mortality endpoints in terms of timeliness and statistical power [12]. This is pertinent for the assessment of MCED screening where trial evidence is not yet available; results from the first RCT of MCED screening in England, the NHS‐Galleri trial, are expected in 2026.

MCED RCT designs with nested mortality outcomes are also increasingly recognised as suitable alternatives to conventional mortality endpoints [13]. Briefly, the nested approach involves retrospective testing of participants in the control arm who develop or die from cancer, comparing their cancer‐specific mortality outcomes with participants in the intervention arm who test positive. This approach focuses on where the clinical benefit of screening actually lies—among test‐positive individuals—and so may provide a more efficient and sensitive approach to evaluating mortality benefit in MCED screening trials.

Other endpoints following cancer diagnosis could also provide early indications of screening benefit or harm and may be valued highly and prioritised by those with cancer. These might include eligibility for curative therapy, reduced treatment side‐effects and morbidity, improved treatment options and response rates, and patient‐focused outcomes that address the physical, psychological, and social aspects of cancer care. These patient‐focused outcomes could be positively impacted by earlier detection and treatment but are historically overlooked and under‐addressed.

Finally, we should also be considering other potentially important changes for the development of new screening innovations. In therapeutics, biopharma companies and regulators (such as the US Food and Drug Administration and UK Medicines and Healthcare products Regulatory Agency) have developed mechanisms to award accelerated or conditional approvals to new innovations that could have a significant impact on outcomes, subject to the delivery of longer‐term or confirmatory evidence at a later stage. If such evidence does not confirm expectations, the innovation can be withdrawn or its implementation modified. As the pace of innovation in screening accelerates, we should be considering analogous approaches for decision‐making bodies such as the USPSTF and UK NSC. For example, screening could be implemented or piloted initially on the basis of agreed intermediate measures, to be later confirmed by nested mortality analyses, and finally by passive follow‐up to detect a mortality change, accounting for other intervening developments such as treatment improvement.

## 3 Conclusion

MCED screening technologies hold great promise for reducing the burden of late‐stage cancers and related cancer mortality, but new approaches are required to evaluate their effectiveness. The decision to implement a national cancer screening programme is based on a combination of evidence from clinical trials, real‐world implementation studies, and health economic modelling, but waiting for RCTs with traditional mortality endpoints can add decades to this process. Innovative trial designs with earlier endpoints that complement cancer mortality are therefore needed to realise the potential benefits of novel screening technologies such as MCEDs more rapidly. Further technological advances in MCED screening that drive down costs would allow these benefits to be realised globally.

## Conflict of interest

The author is an employee of GRAIL, Inc, a Member and former Chief Executive of Cancer Research UK, and a member of the President's Advisory Council of the European Association of Cancer Research.
